# Potentially long-lasting effects of the pandemic on scientists

**DOI:** 10.1038/s41467-021-26428-z

**Published:** 2021-10-26

**Authors:** Jian Gao, Yian Yin, Kyle R. Myers, Karim R. Lakhani, Dashun Wang

**Affiliations:** 1 Center for Science of Science and Innovation, Northwestern University, Evanston, IL 60208 USA; 2 Kellogg School of Management, Northwestern University, Evanston, IL 60208 USA; 3 Northwestern Institute on Complex Systems, Northwestern University, Evanston, IL 60208 USA; 4 McCormick School of Engineering, Northwestern University, Evanston, IL 60208 USA; 5 Harvard Business School, Harvard University, Boston, MA 02163 USA; 6 Laboratory for Innovation Science at Harvard, Harvard University, Boston, MA 02134 USA; 7 Institute for Quantitative Social Science, Harvard University, Cambridge, MA 02138 USA

**Keywords:** Scientific community, Careers

## Abstract

Two surveys of principal investigators conducted between April 2020 and January 2021 reveal that while the COVID-19 pandemic’s initial impacts on scientists’ research time seem alleviated, there has been a decline in the rate of initiating new projects. This dimension of impact disproportionately affects female scientists and those with young children and appears to be homogeneous across fields. These findings may have implications for understanding the long-term effects of the pandemic on scientific research.

## Abstract (web summary)

The pandemic has caused disruption to many aspects of scientific research. In this Comment the authors describe the findings from surveys of scientists between April 2020 and January 2021, which suggests there was a decline in new projects started in that time.

The COVID-19 pandemic has disrupted the scientific enterprise^1–3^. Researchers in the “bench” sciences, female scientists, and those with young children experienced significant declines in research time and other publication-based metrics, according to data collected before the summer of 2020 (refs. ^1–8^). Now, more than a year into the pandemic and with multiple vaccines developed, circumstances have evolved substantially. This raises an important question: has the pandemic’s impact on scientists evolved as well?

To answer this question, we distributed a survey in January 2021 by randomly sampling US- and Europe-based scientists across a wide range of scientific fields. Importantly, we adopted the same sampling strategy as a previous survey we conducted in April 2020 (ref. ^1^), which allowed us to directly compare the results of the surveys at these two very different stages of the pandemic (Supplementary Note 1 and Supplementary Fig. 1). In the January 2021 survey, we asked scientists many of the same questions from the April 2020 survey, including professional and demographic features. We also added new questions that compare their overall research activity and output in 2020 with 2019, including the number of new research publications, new submissions, new collaborators, and new research projects they started each year. Furthermore, we asked scientists whether or not they conducted any COVID-19-related research in 2020. In total, we collected responses from 6982 respondents across the two surveys who self-identified as faculty or principal investigators (Supplementary Note 2). To supplement our survey findings, we also conducted a series of analyses using a large-scale publication dataset, the Dimensions database, which captures both articles and preprints published up to the beginning of 2021.

## The pandemic’s impact on scientists has changed

**Fig. 1:**
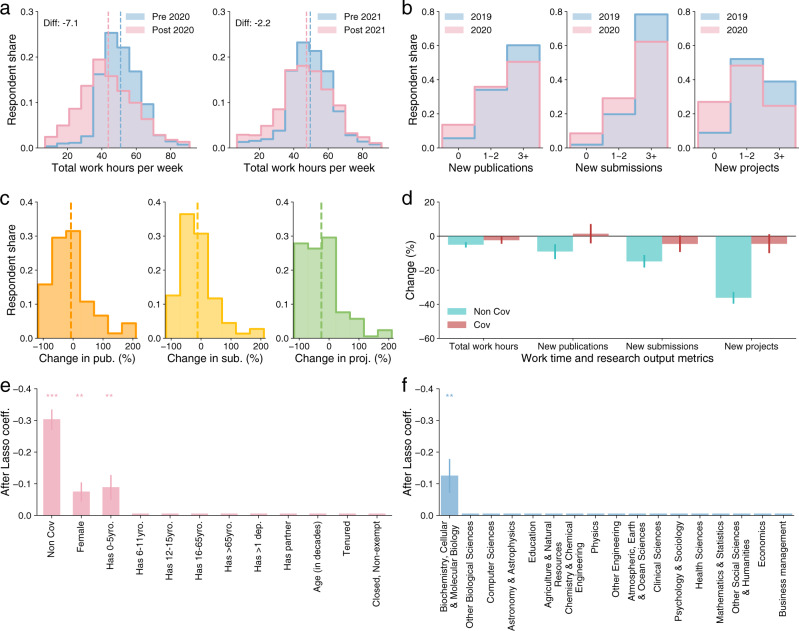
Gradual recovery of total work time and substantially fewer new research projects. **a** The distributions of total work hours per week for the pre- and post-periods. The left and right panels correspond to the surveys in April 2020 and January 2021, respectively. Vertical dashed lines mark the means, and the difference in means is shown. **b** The distributions of new publications, new submissions, and new projects for 2019 and 2020. Reported values are categorized into three bins. **c** The distributions of the changes in new publications, new submissions, and new projects in 2020 relatively to 2019. Changes over 200% are set as 200%. Vertical dashed lines mark the means. **d** The average change in work time and output metrics, unpacked by whether scientists have worked on COVID-19-related topics in 2020. Error bars indicate 95% confidence intervals. **e** Regression analysis of the change in new research projects. The Lasso regression selects professional and demographic features most predictive of the declines in new projects after controlling for research fields. The regression also includes a COVID-19 dummy variable capturing whether the respondent reported engaging with COVID-19-related research in 2020. **f** The Lasso regression selects field features most predictive of the declines in new projects after controlling for demographic factors and the non-COVID-19 dummy. Error bars indicate standard errors, and stars indicate significant levels: *p < 0.1, **p < 0.05, ***p < 0.01.

During the early phase of the pandemic, scientists reported a sharp decline in time spent on research^1,2,6^. For example, in April 2020, scientists reported an average decrease of 7.1 h per week compared to pre-pandemic levels (Fig. 1a, left). In January 2021, however, scientists reported only minor differences between their current and pre-pandemic total work time (Fig. 1a, right). Total work hours in January 2021 were still lower than the pre-pandemic levels, but the difference was only 2.2 h per week on average. In percentage terms relative to pre-pandemic levels, the impact on total work hours changed from roughly −14% in April 2020 to −4% in January 2021 (Supplementary Fig. 2), suggesting that some recovery had occurred.

Focusing on publication metrics, the average self-reported number of new publications or submissions in 2020 was only moderately lower than in 2019 (Fig. 1b, left and middle). These reported changes are consistent with measurements from publication databases (Supplementary Fig. 10) and prior studies^8,9^, offering further signs of recovery. Yet, as we show next, these metrics mask an important way in which the pandemic affected scientists: the rate of new research projects initiated.

## Fewer new projects initiated during the pandemic

Only about 9% of scientists reported that they initiated zero new research projects in the year of 2019, but this fraction increased roughly threefold in 2020 to about 27% (Fig. 1b, right). Figure 1c plots the distributions of individual-level changes in the number of new publications, new submissions, and new projects. The changes in new publications (Fig. 1c, left) and new submissions (Fig. 1c, middle) were rather modest compared to the large negative change in new projects (Fig. 1c, right). These patterns are significant whether changes are measured in absolute or relative values (Supplementary Figs. 3–4).

Roughly one-third of survey respondents reported working on COVID-19-related research in 2020 (Supplementary Note 9), echoing science’s strong response to the pandemic^10–12^. However, this suggests that the decline in new projects we observe may be even larger among respondents who did not pursue COVID-19-related research. Indeed, when we separate our sample based on whether or not the scientist reported working on any COVID-19-related research, we find that the two groups show substantially different patterns (Fig. 1d). Scientists who worked on COVID-19-related research reported almost no changes in any of the productivity metrics, compared with pre-pandemic levels. By contrast, the scientists who reported not working on COVID-19 reported significantly larger decreases in total work time (−5%), new publications (−9%), new submissions (−15%), and new projects (−36%). In absolute terms, the decline in new research projects corresponds to the loss of one new project per scientist in 2020 (Supplementary Fig. 3). This decline seems rather meaningful given that scientists in our sample reported initiating only about three new projects in a normal year. When we examine these measures across the various stages of scientific production, shifting from a focus on finished papers to starting new projects, it appears that the impact of the pandemic is increased earlier in the research pipeline (Fig. 1d). These observations further suggest that the pandemic’s long-lasting effects on scientific productivity loom large on the horizon.

## Field- and group-level differences

How does the decline in new research projects vary across various professional and demographic characteristics? To answer this question, we employ a Lasso regression approach to select features most predictive of changes in new projects (Supplementary Note 3). First, we examine demographic features, after controlling for scientific fields and a dummy variable indicating whether the respondent reported engaging with COVID-19-related research. The features associated with the largest declines in new projects are being a female or having young children (Fig. 1e). Notably, these are the same groups of scientists who reported the largest initial disruptions to their research in the early phase of the pandemic^1,2,5^, suggesting the loss of new projects may further exacerbate the pandemic’s already highly unequal effects on scientists, especially for those who did not pursue COVID-19-related research in 2020.

We next focus on differences in the rate of starting new projects across scientific fields. We again employ a Lasso regression model, this time selecting indicator variables for fields while controlling for demographic features and the COVID-19-related research indicator variable. We find that, overall, the declines in new projects appear homogeneous across fields (Fig. 1f). While all fields reported declines in starting new projects, only biochemists reported significantly lower-than-average declines (post-Lasso regression coefficient *b* = −0.12, *S.E.* = 0.05, *P* value = 0.02) after controlling for other individual-level features. This sharply contrasts with the heterogeneity observed across fields at earlier stages of the pandemic^1^. Given that the pandemic has limited access to lab facilities or travels to field sites, the level of homogeneity observed here is rather unexpected. Indeed, despite the apparently different nature of work across fields, no scientific fields were immune to the reduced number of new projects, further suggesting that this decline are likely due to factors that are common across fields. This finding of homogeneity persists using several alternative measures (Supplementary Note 7).

## Decreases in new co-authorships

**Fig. 2:**
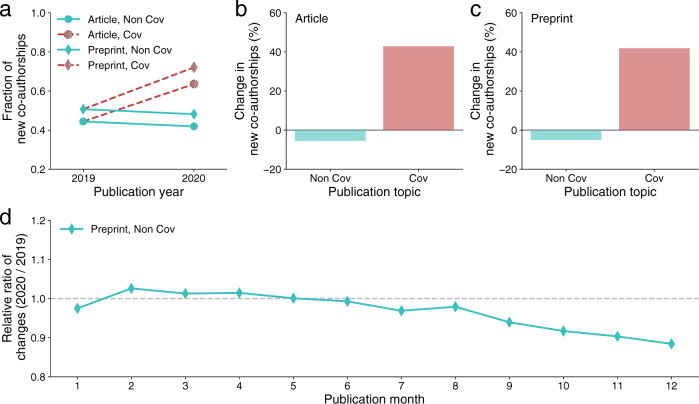
Changes in new co-authorships measured by large-scale publication datasets. **a** The fraction of new co-authorship pairs in the author list of COVID-19 and non-COVID-19 papers published as articles or preprints in 2019 and 2020. **b** The average change in the fraction of new co-authorships measured for articles in 2020 comparing with that in 2019. **c** The average change in the fraction of new co-authorships measured for preprints in 2020 comparing with that in 2019. **d** The relative ratio of the fraction of new co-authorships in 2020 over the fraction in 2019 measured for non-COVID-19 preprints published in each month of the year.

Given the long gestation time for new research ideas to mature and be published^13^, the decline in new research projects suggests the impact of the pandemic may not manifest in the publication record for years. Nonetheless, one metric where we might begin to observe some signal is the rate of new co-authorships. Indeed, the pandemic and associated social distancing measures halted many in-person interactions that might otherwise have facilitated the flow of new research ideas and collaborations^14–16^. To this end, we examine changes in the rate of new co-authorships using a large-scale publication dataset that includes about 9.5 million articles and preprints published in 2019 and 2020. Specifically, we examine the rate of new co-authorships for both COVID-19 and non-COVID-19-related papers by calculating the fraction of new author pairs^17,18^ (Supplementary Note 5). For both articles and preprints, we find that the fraction of new co-authorships appearing on COVID-19-related papers increased in 2020 (Fig. 2a) by roughly 40% compared to the 2019 level (Fig. 2b, c), which is largely consistent with prior studies^19,20^. By contrast, new co-authorships on non-COVID-19-related papers exhibited markedly different patterns (Fig. 2a), showing a significant decrease of roughly 5% compared to the 2019 level (Fig. 2b, c). These estimates from the publication record are broadly consistent with the self-reported changes in new collaborators in our survey (Supplementary Fig. 9).

The decrease in the rate of new co-authorships for non-COVID-19 papers published in 2020 may seem unexpected, given that many of these collaborations may have started before the pandemic, suggesting the effect may be even stronger for collaborations that started later in time. To test this hypothesis, we focus on non-COVID-19-related preprints published in 2020. Given the time required for publication in peer-reviewed journals, one might expect the decrease in new co-authorships is more pronounced in preprints than in published articles, and the effect might grow stronger over the course of 2020 as the pandemic unfolded. To test these predictions, we plot the temporal trends in the rate of new co-authorships for non-COVID-19 preprints published in 2020 relative to those published in 2019 (Fig. 2d). We find that the decline in new co-authorships is more evident for those published in the second half of the year than the first half, suggesting the effect is especially pronounced for projects finished later in the year. Note that the observed patterns in new co-authorships may flow from other social and institutional factors. Nonetheless, our survey data show that the rates of new collaborators and new projects are strongly correlated with each other even after controlling for other factors (Supplementary Tables 1 and 2). Overall, these results provide supporting evidence consistent with the hypothesis that the rate of starting new projects on non-COVID-19-related research has declined during the pandemic.

## Concluding remarks and discussion

Taken together, our surveys and analyses reveal two important patterns. The first suggests some optimism: the amount of time scientists are spending on their research has almost returned to pre-pandemic levels, and most publication-based metrics show only minor declines. On the other hand, our analyses suggest that, even though scientists are returning to work, they have been substantially less likely to pursue new research projects. This suggests that the impacts of the pandemic on science may be longer-lasting than is commonly imagined.

These findings are important for several reasons. While many studies have focused on scientists who pivoted their research towards the pandemic^12^, it is important to recognize that the majority of scientists did not carry out COVID-19-related research (Supplementary Note 9), and it is this majority who appear especially disrupted. Paper submissions and publications appear to be holding steady, if not on the rise^8,9^. However, the finding that researchers pursued fewer new projects in 2020 suggests that these trends may reflect scientists working on established topics, writing up existing research, submitting drafts earlier than they would have otherwise^3^, writing more grant proposals than typical^21^, or revisiting old data and reviving legacy projects that they would not have pursued otherwise. While the impact of these changes remains unclear, they suggest that publication trends alone may paint an incomplete picture of the productivity of the research enterprise.

While the decline in new research projects coincides with the decrease in new co-authorships, many other factors may also play a role. Some of the potential mechanisms include decreased access to facilities and field sites, a decline in in-person training and mentorship, less funding or support for non-COVID-19-related research, increased teaching demands such as redesigning courses, the psychological toll caused by the pandemic^22,23^, or uncertainty about how the pandemic will unfold in the coming months and years. The homogeneous nature of the decline in starting new projects across fields, however, suggests that the primary reasons for this decline may not be unique to the nature of work in any particular field but are instead more common to all scientists.

Overall, these findings have important implications for science policy. First, they are consistent with face-to-face interactions and collaborations being an important channel for new ideas^14–16^, reinforcing the value of resuming in-person activities. While there could be substantial gains from certain aspects of science shifting online (e.g., virtual seminars reducing travel demands and bridging geographical gaps)^24^, it remains unclear how well virtual tools can facilitate important social functions related to the formation of new ideas. Second, these results may contribute to current policy discussions aimed at encouraging social interactions, facilitating new collaborations^14^, or promoting new ideas (e.g., institutional bridge funds^24^). Ultimately though, successful rebuilding of the global research enterprise would also depend on how well policy makers and institutional leaders address and manage the mental-health challenges facing scientists^23^.

The decline in pursuing new projects is particularly pronounced for women or caregivers of young children, which is consistent with related work^1,2,5,25,26^. Likely in response to these sorts of patterns, many institutional leaders implemented policies such as tenure clock extensions^24^. As institutions begin their phased return, it may be tempting for decision makers to evaluate short-term metrics to gauge research outputs and inform their subsequent policies. Yet, our results suggest that these short-term metrics may mask long-lasting effects of the pandemic. It is also important to recognize that even as universities reopen, children under the age of twelve remain ineligible for COVID-19 vaccines at the time of this writing, which has further implications for scientists with young children. Ignoring these long-run consequences may have profound implications not just for the inequality of science but also its long-term vitality^27–29^. At the same time, it also suggests that short-term investments, such as childcare support, may yield long-term benefits.

Our analyses have several limitations. (1) Our two surveys span only US- and Europe-based institutions, which limits the geographic coverage of our analysis. Yet, our preliminary analyses suggest that low-income or developing countries appear to experience substantially larger declines in new co-authorships on non-COVID-19-related research (Supplementary Fig. 13). Given the global disparity in the pandemic^11,30^, expanding our analyses to other regions would be extremely valuable. (2) Our survey respondents are from self-selected samples and may not be representative of the full population of scientists. In particular, those who felt strongly about sharing their situation may be more likely to respond. (3) Although the survey results and actual research outputs show a high degree of consistency (Supplementary Fig. 5), as with any survey, there may be biases in the self-reported metrics. Similarly, measurements using publication records may be limited by the fact that new co-authorships, especially those on non-COVID-19 topics, may take longer to come to fruition. (4) The number of new projects is a relatively new measure, and may have been interpreted differently by scientists from different backgrounds. As such, continued work investigating the value and reliability of this metric is important and could further enrich our understanding of early-stage research. (5) Our surveys do not capture health information, preventing us from controlling for scientists’ direct or indirect exposure to the virus. (6) The effects discussed in this paper are based on correlations, leaving open questions about what exactly may be the key mechanisms causing the decline in new research projects.

Taken together, our findings suggest a potentially long-lasting effect of the pandemic on scientists that has thus far received little attention: a decrease in initiating new research projects. This dimension of impact appears to be rather homogeneous across fields and affects disproportionately female scientists and those with young children. Thus it is vital for science funders and institutional leaders to pay attention to the long-term effects of the pandemic on the scientific enterprise—even when science might appear to be recovering from its initial disruptions.

### Reporting summary

Further information on research design is available in the Nature Research Reporting Summary linked to this article.

## Supplementary information

Supplementary Information

Reporting summary

## Data Availability

Because of the sensitive nature of some variables collected by the surveys, the IRB-approved protocol does not permit individual-level data to be made unrestricted and publicly available. Researchers interested in obtaining restricted, anonymized versions of this individual-level data should contact the authors to inquire about obtaining an IRB-approved institutional data sharing agreement. This work also uses data sourced from Web of Science and Dimensions.ai. Researchers who wish to access raw data should contact the data sources directly.

## Code availability

Code necessary to reproduce all plots and statistical analyses are freely available at http://kellogg-cssi.github.io/long_covid and Figshare 10.6084/m9.figshare.16528557.
