# Supplementary material for: Potentially long-lasting effects of the pandemic on scientists
Source: Nat Commun. 2021 Oct 26;12:6188. doi: 10.1038/s41467-021-26428-z (PMC8548590; doi:10.1038/s41467-021-26428-z)
Supplement: Supplementary file 1 — Supplementary Information [file 41467_2021_26428_MOESM1_ESM.pdf]

Supplementary Information

Potentially long-lasting effects of the pandemic on scientists

Jian Gao[1,2,3], Yian Yin[1,3,4], Kyle R. Myers[5,6], Karim R. Lakhani[5,6,7], Dashun Wang[1,2,3,4*]

[1] Center for Science of Science and Innovation, Northwestern University, Evanston, IL 60208, USA
[2] Kellogg School of Management, Northwestern University, Evanston, IL 60208, USA
[3] Northwestern Institute on Complex Systems, Northwestern University, Evanston, IL 60208, USA
[4] McCormick School of Engineering, Northwestern University, Evanston, IL 60208, USA
[5] Harvard Business School, Harvard University, Boston, MA 02163, USA
[6] Laboratory for Innovation Science at Harvard, Harvard University, Boston, MA 02134, USA
[7] Institute for Quantitative Social Science, Harvard University, Cambridge, MA 02138, USA
[*] Correspondence to: dashun.wang@northwestern.edu

Table of Contents

Supplementary Note 1. Survey Sampling and Recruitment 2
1.1 Web of Science corresponding authors 2
1.2 Participant recruitment 2
Supplementary Note 2. Survey Instrument and Sampling Approach 3
2.1 Survey questions 3
2.2 Research field definitions 4
2.3 Survey data sampling approach and basic statistics 5
Supplementary Note 3. Covariate Selection and Regression Approach 5
3.1 Lasso selection and post-Lasso regression 5
3.2 Ordinary Least Squares (OLS) and probit regression 6
Supplementary Note 4. Publication Data and Matching Approach 6
4.1 The Dimensions publication data 6
4.2 Matching survey respondents to Dimensions authors 7
Supplementary Note 5. Measuring the Rate of New Co-authorships 7
5.1 New co-authorships based on publication data 7
Supplementary Note 6. Changes in Time and Output Metrics 7
6.1 Changes in total work hours 7
6.2 Changes in publication, submission, and project 8
6.3 Comparison of publication data in survey and Dimensions 8
Supplementary Note 7. Changes in Projects and Collaborators by Survey 9
7.1 Group-level and field-level changes in new projects 9
7.2 Robustness checks using alternative measures 9
7.3 Changes in new collaborators 9
7.4 Associations between new projects and new collaborators 10
Supplementary Note 8. Results based on the Dimensions Publication Data 10
8.1 Trends of total and average publications per year 10
8.2 Changes in new co-authorships measured by publication records 11
Supplementary Note 9. Survey Data on COVID-19 Research 12
9.1 COVID-19-related research by survey data 12
Supplementary Figures 13
Supplementary Tables 22
Supplementary References 24

Supplementary Note 1. Survey Sampling and Recruitment

1.1 Web of Science corresponding authors

The Web of Science (WoS) publication database is leveraged to compile a large, plausibly random list of active scientists. We rely on the WoS database for two major reasons: (1) it is one of the most authoritative and widely used large-scale publication and citation corpuses available[1-5]; (2) it provides systematic coverage of corresponding author email addresses. Here, we attempt to focus on scientists that are likely to be in a more stable research position and still be active. Starting from about 21 million WoS papers published in the period of 2010-2019, we do an initial filtering based on the publication venue. Specifically, we exclude papers published in journals that are ranked to bottom 25% by the impact factor at the time (WoS Journal Citation Reports) for its WoS-designated category. We then extract all email addresses associated with these papers and consider an email address as a potential participant if (1) it is associated with at least two papers in the corpus, and (2) its affiliation of the most recent paper is based in the U.S. or Europe.

After this data filtering process, we are left with approximately 1.5 million unique email addresses, with about 521,000 in the U.S. and about 938,000 in Europe. We then randomly shuffled the two lists of email addresses separately and sampled 280,000 from the U.S. and 200,000 from Europe for the April 2020 survey[6]. As a part of a broader outreach strategy underlying this and other research projects, we oversampled the U.S. in comparison with Europe. Furthermore, from remaining email addresses in the two lists, we randomly sampled 140,000 from the U.S. and 100,000 from Europe for the January 2021 survey.

1.2 Participant recruitment

We recruited participants by sending invitations to the sampled email addresses. We followed the same recruitment process across the two surveys and used personalized texts to accommodate the time of the surveys. The recruitment email text used in the January 2021 survey is as follows:

> *Dear [Author Name],*
>
> *We need your help to shed light on how the coronavirus pandemic is affecting scientists like you. This study builds on our previous research published in Nature Human Behavior with Kyle R. Myers and Karim R. Lakhani at the Laboratory for Innovation Science at Harvard.*
>
> *Please take a brief moment to complete this short 5-minute survey as part of a research study. Your responses will help scientists and policymakers understand and respond to this rapidly evolving situation. The study protocol has been approved by Northwestern University's Institutional Review Board (IRB, STU00212699).*
>
> *Click HERE [hyperlink] to complete the survey or, copy and paste the URL below into your internet browser:*
> *[link]*
>
> *Thank you for your time!*
>
> *Sincerely,*
>
> *Dashun Wang, Ph.D.*
> *Northwestern Kellogg Center for Science of Science & Innovation (CSSI)*

Supplementary Note 2. Survey Instrument and Sampling Approach

2.1 Survey questions

The April 2020 survey includes questions on demographic information (age, gender, cohabitation, dependents), professional information (position type, institution type, fields of study, type of research, tenure status), and time allocation (time spent on different activities before and after the pandemic)[6]. In the January 2021 survey, besides retaining these questions, we added new questions about whether respondents worked on topics related to the coronavirus in 2020 and how their research output metrics changed in 2020 compared with 2019. Respondents were not required to answer any of the demographic questions. The January 2021 survey questions underlying the variables used in our analyses are as follows:

Q. Which of the following best describes your current position?
- *Faculty or principal investigator | Research staff or assistant | Post-doctoral researcher | Graduate student in a doctoral program | Retired faculty or principal investigator still engaged in research | Retired scientist no longer engaged in research | Other*

Q. Which of the following best describes your field of study?
- *[list of 20 fields]*

Q. Which of the following best describes the institution you are primarily affiliated with?
- *University or college | Non-profit research organization | Government or public agency | For-profit firm | Other*

Q. Please answer the following:
- *Is your institution physically closed to non-essential personnel?*
    - *Yes | No | Not relevant*
- *Are you exempt from the closure and allowed to travel to your work site(s)?*
    - *Yes | No | Not relevant*
- *Do you have tenure?*
    - *Yes | No | Not relevant*

Q. Gender:
- *Male | Female | Other | Prefer not to say*

Q. Age:
- *Under 20 | 20-24 | 25-29 ... 75-79 | 80 or older | Prefer not to say*

Q. Number of dependents of any age you care for:
- *0 | 1 | 2 | 3 or more | Prefer not to say*

Q. In what age group(s) are your dependents? Note. You may select multiple
- *0-2 years old | 3-5 years old | 6-11 years old | 12-18 years old | 19-65 years old | Over 65 years old*

Q. Cohabitation status:
- *I reside with a partner, spouse, or significant other | I reside with friends | I reside by myself | Other | Prefer not to say*

Q. Around this time last year (January 2020), about how many hours per week did you work on anything related to your job? (e.g., researching, teaching, writing)

- *14-21 hours per week (avg. 2-3 hours every day) | 21-28 hours per week (avg. 3-4 hours every day) |... | 77-84 hours per week (avg. 11-12 hours every day) | More than 84 hours per week (avg. 12 hours or more every day)*

Q. Currently (January 2021), about how many hours per week are you working? (e.g., researching, teaching, writing)

- *14-21 hours per week (avg. 2-3 hours every day) | 21-28 hours per week (avg. 3-4 hours every day) | ... | 77-84 hours per week (avg. 11-12 hours every day) | More than 84 hours per week (avg. 12 hours or more every day)*

Q. During the year 2020, have you work on research topics related to this COVID-19 pandemic?

- *No | Yes*

Q. During the year 2019, how many new research projects did you start? Please write a number.

- *[0, 1, 2, ..., 100]*

Q. During the year 2020, how many new research projects did you start? Please write a number.

- *[0, 1, 2, ..., 100]*

Q. During the year 2019, with how many people did you establish new collaborations? Please write a number.

- *[0, 1, 2, ..., 100]*

Q. During the year 2020, with how many people did you establish new collaborations? Please write a number.

- *[0, 1, 2, ..., 100]*

For the following, please consider your "research publications" as all of your publications that focus on a research question. (e.g., journal articles, conference proceedings, patents, books. Ignore commentary, editorials, etc.)

Q. During the year 2019, how many research publications did you submit (including journals/conferences/preprint servers)? Please write a number.

- *[0, 1, 2, ..., 100]*

Q. During the year 2019, how many peer-reviewed research publications did you publish? Please write a number.

- *[0, 1, 2, ..., 100]*

Q. During the year 2020, how many research publications did you submit (including journals/conferences/preprint servers)? Please write a number.

- *[0, 1, 2, ..., 100]*

Q. During the year 2020, how many peer-reviewed research publications did you publish? Please write a number.

- *[0, 1, 2, ..., 100]*

2.2 Research field definitions

Research fields in our survey are built on the field classifications used in national surveys such as the U.S. Survey of Doctorate Recipients (SDR) with an aggregation to ensure sufficient sample

sizes within each field. We made additions to the fields by including Business Management, Education, Communication, and Clinical Sciences, as they reflect major schools at most universities and/or did not immediately map to some of the default fields used in the SDR, for example, medical specialties are not included in the "Health Sciences" field in SDR.

2.3 Survey data sampling approach and basic statistics

After a total of 480,000 emails sent in April 2020, there are 8447 individuals that entered the survey and continued past the consent stage. For our analysis, we focus entirely on responses from faculty or principal investigators (PIs). Thereby, we retain respondents who self-identified as "Faculty principal investigator" or "Retired faculty or principal investigator still engaged in research" and reported working for a "University or college", "Non-profit research organization", "Government or public agency", or "Other" (excluding whose who reported working for a "For-profit firm"). We further drop observations that have missing data for key variables (working time, age, gender, and field of study). We do not impute missing variables as it may introduce unnecessary noise[7]. Altogether, these criteria lead to a sample of 4535 respondents used in the analyses for the April 2020 survey.

Following a similar procedure, we sent out our January 2021 survey to 240,000 email addresses, and 4672 individuals entered the survey and continued past the consent stage. We focused on responses from faculty or principal investigators (PIs) and dropped observations that have missing data for working time. These criteria lead to a base sample of 2447 respondents from the January 2021 survey in our analysis. We further drop missing data for research output metrics (i.e., projects, collaborators, submissions, and publications) and key variables (e.g., age, gender, field of study, tenure status, etc.) during the analyses involving them.

We adopted the same sampling strategy for the April 2020 survey and the January 2021 survey. To quantify possible bias in our sample of respondents, here we compare the publication rates of respondents as well as non-respondents in the two surveys. Specifically, we link survey respondents back to the WoS database using their charity email addresses (2533 respondents in the 2020 survey) and personalized survey links associated with their email addresses (2411 respondents in the 2021 survey). Then, for each linked respondent, we calculate the number of publications in the periods of 2019, 2018-2019, 2016-2019, and 2012-2019 based on the WoS database. We repeat this practice for non-respondents. We find that there is no significant difference between respondents in the 2020 survey and those in the 2021 survey in terms of publication rates (Supplementary Figure 1a), or between non-respondents in these two surveys (Supplementary Figure 1b). These results offer support for our randomization strategy on sampling email addresses, allowing us to directly compare data obtained from the two surveys.

Supplementary Note 3. Covariate Selection and Regression Approach

3.1 Lasso selection and post-Lasso regression

We use multivariate regressions to explore whether changes associated with a group of individuals change after conditioning on other observables (e.g., the demands of home life unique to certain individuals, or the nature of work in certain research fields). We select a set of important covariates (or transformations thereof) that should be included in regressions by employing a Lasso method, which provides a data-driven approach to this selection problem by excluding covariates from the regression that do not improve the fit of the model[8,9]. Specifically, our Lasso approach is to include

a vector of indicator variables for the research fields and the professional and demographic groups of interest. When focusing on field-level differences, we include the professional and demographic variables in the control set. In turn, when focusing on professional and demographic-level differences, we include field variables in the control set. To make minimal assumptions about the functional form of control variables, we conduct the following transformations to expand the set of controls: for all continuous variables we use inverse hyperbolic sine (which approximates a logarithmic transformation while allowing zeros), square and cubic transformations, and we interact all indicator variables with the linear versions of the continuous variables.

The Lasso selection approach is performed using the lasso linear package in Stata 16 © software. We use the defaults for constructing initial guesses, tuning parameters, number of folds (ten), and stopping criteria. We use the two-step cross-validation "adaptive" Lasso model where an initial instance of the algorithm is used to make a first selection of variables, and then a second instance occurs using only variables selected in the first instance. The variables selected after this second run are then used in a standard post-Lasso Ordinary Least Squares (OLS) regression with heteroskedastic robust standard errors.

3.2 Ordinary Least Squares (OLS) and probit regression

A standard OLS regression model is used to explore the relationship between two variables of interests, in our analysis, the association between the change in new projects and the change in new collaborators. We add a set of control variables in the OLS regressions with robust standard errors, including both professional and demographic variables and the dummy of research fields. Moreover, we employ a probit regression model to study the how these variables are associated with the probability for scientists to work on COVID-19-related research. The dependent variable is a dummy variable that takes 1 if scientists reported working on COVID-19-related research in 2020 and 0 if otherwise. The independent variables include professional and demographic variables as well as the field dummy.

Supplementary Note 4. Publication Data and Matching Approach

4.1 The Dimensions publication data

For the database of scientific publications, we use Dimensions[10], a data product by Digital Science, which provides a systematic coverage of research papers and preprints. The Dimensions data is updated in a timely manner with relatively smaller time lags compared with other alternative large-scale publication datasets, offering an opportunity to study recent publishing trends. In March 2021, we retrieved from the Dimensions database papers and preprints published up to the end of 2020. For each paper, we obtain information on its title, author list, publishing venue, publication date, fields of study, DOI (Digital Object Identifier), and publication type (e.g., articles and preprints). Authors in the corpus have been pre-disambiguated by Dimensions.

We also construct a set of publications that are related to the COVID-19 pandemic by leveraging the Dimensions searching engine. Specifically, we follow prior work[11] and search for papers published in 2020 using the following query suggested by Dimensions[12]:

"2019-nCoV" OR "COVID-19" OR "SARS-CoV-2" OR "HCoV-2019" OR "hcov" OR "NCOVID-19" OR "severe acute respiratory syndrome coronavirus 2" OR "severe acute respiratory syndrome corona virus 2" OR (("coronavirus" OR "corona virus") AND (Wuhan OR China OR novel))

This searching process yields in total 216,187 COVID-19-related papers published in 2020 out of all papers indexed by the Dimensions database.

4.2 Matching survey respondents to Dimensions authors

We link the respondents of January 2021 survey to authors in Dimensions. For each respondent, we first build a list of papers that are associated with the respondent's email address in the WoS dataset and collect each paper's DOI, one of the most commonly used identifiers for scientific publications. Then, we retrieve papers from Dimensions using the list of DOIs and collect author information for each paper. Next, we aggregate the author information and identify the Dimension author that can be matched to the respondent on both first and last names. Using this matching process, we linked 2141 out of all 2447 respondents in the January 2021 survey to Dimensions authors, yielding a matching rate of about 87%. For these matched respondents, we further calculate the number of their publications every year in Dimensions.

Supplementary Note 5. Measuring the Rate of New Co-authorships

5.1 New co-authorships based on publication data

To investigate the temporal changes in collaborations among scientists, we extract collaboration patterns from the Dimensions publication data and calculate a measure of new co-authorships. Specifically, building on rich literature of team science[5,13-17], we define the rate of new co-authorships as the fraction of author pairs that have not co-authored previously to all authors pairs on a paper. Considering that the baseline rate of new co-authorships may change as team size (i.e., the number of co-authors on a paper) increases, here we only focus on teams with 50 or less authors[18]. We repeat our analysis for narrow ranges of team sizes as additional robustness checks (Supplementary Figure 12).

To construct a comprehensive record of previous co-authorships, we extracted all disambiguated author pairs from publications in Dimensions since 1950. For each author pair, we record their earliest year of co-authorship (i.e., the time of their first co-authored publication). Then, we iterate over all co-author-paper combinations, classifying each co-author pair as an "old" co-authorship if their earliest co-authorship year is before the publication year of this paper. For example, given a paper published in 2020 with three authors, Alice, Barbara and Cindy, if Alice has published one paper with Barbara in 2019 and another paper with Cindy in 2018, but Barbara and Cindy haven't co-authored any paper before 2020, the fraction of new co-authorships in this paper is 1/3. We also use information on publication month as well as publication types for our further analysis.

Supplementary Note 6. Changes in Time and Output Metrics

6.1 Changes in total work hours

We investigate the change in work time (comparing post-pandemic levels with pre-pandemic levels) and explore how the magnitude of changes shits over time as the pandemic unfolds in 2020. Supplementary Figure 2 shows the changes in total work hours reported by respondents in April 2020 survey and January 2021 survey, which are 9 months apart. We find that the total work hours per week in the post pandemic period increases from an average of about 44 hours in April 2020 to about 47 hours in January 2021 (Supplementary Figure 2a), largely narrowing the gap between pre and post pandemic work hours. The distribution of changes in total work hours at the individual

level in April 2020 exhibits a clear shift to the left, with a mean decrease of about 7 hours. By comparison, the distribution in January 2021 becomes more symmetric, showing only a minor decrease of about 2 hours on average (Supplementary Figure 2b). The percentage change in work hours has also increased from about -14% to about -4%, again suggesting a clear recovery pattern in working time (Supplementary Figure 2c).

6.2 Changes in publication, submission, and project

We calculate the changes in research output metrics reported by respondents in the January 2021 survey. Supplementary Figure 3 shows the average values and the relative changes in the number of new research publications, new submissions, and new projects. We find that research outputs in 2020 are on average less than those in 2019 across all three metrics (Supplementary Figure 3a). More specifically, the average number of new publications, submissions and projects has decreased by 0.4 (from 4.5 in 2019 to 4.1 in 2020), 0.9 (from about 6.2 in 2019 to 5.3 in 2020) and 0.7 (from about 2.5 in 2019 to 1.8 in 2020) approximately. In a relative term, the decline in new projects (-26%) is more pronounced than in new submissions (-5%) and publications (-11%) (Supplementary Figure 3b). These results are largely robust when calculating changes using a logged value (Supplementary Figure 3cd), where we use the form log(x+1) to deal with 0 in the raw value x.

For the calculation of relative changes (Supplementary Figure 3cd), a small fraction (about 3-5%) of respondents reported a zero value for 2019 but a non-zero for 2020 are excluded from the analyses because the denominator can't be zero. To test the robustness of our results, we also calculate the absolute changes in the number of new publications, new submissions, and new projects comparing 2020 with 2019 (Supplementary Figure 4). We find that all the three research output metrics show a negative change on average, providing additional support to our results.

6.3 Comparison of publication data in survey and Dimensions

To test whether the self-reported number of publications is aligned with that tracked by other data sources, here we calculate publication counts for respondents matched in the Dimensions publication database (Supplementary Note 4). For each of those with matched survey respondents, we calculate the number of articles published in 2019 and 2020 and compare with the number reported in the survey (Supplementary Figure 5). We find that our survey data is strongly and positively correlated with the Dimensions data, showing a Spearman's rank correlation of 0.71 for 2019 (Supplementary Figure 5a) and 0.75 for 2020 (Supplementary Figure 5b). A linear fit of the two data can explain about 57% of variances, indicating an overall well alignment. Notably, the number of publications by Dimensions tends to be larger than the survey-reported number especially for more productive respondents, which may be explained by several reasons, such as recalling biases when respondents published a large number of papers and cognitive biases as respondents may have different perceptions of authorship when there are many co-authors on a single paper. As robustness checks, we also examine the relationship based on logged values, where we use $\log(x+1)$ to avoid 0 in the raw value x. We find that these positive correlations are very robust when using logged values (Supplementary Figure 5cd).

Supplementary Note 7. Changes in Projects and Collaborators by Survey

7.1 Group-level and field-level changes in new projects

We explore the differences in the decline of new research projects in 2020 relative to 2019 across demographic variables and scientific fields. Supplementary Figure 6 shows the average changes in new projects, aggregated by professional and demographic variables as well as fields. We find that scientist who haven't pursue COVID-19-related research reported a much larger declines in new projects, showing a change of about -57%, compared with the overall sample average of about -27% (Supplementary Figure 6a). Female scientists and those with young dependents are also affected more than others. In addition, we find that scientists in disciplines that rely on physical laboratories—such as biochemistry, biology, and astronomy—reported the largest loss of new ideas in 2020, in a range of 38-44% below the 2019 level (Supplementary Figure 6b). By comparison, scientists in social science fields (e.g., business, economics, and humanities) reported smaller declines in new ideas in 2020 compared with 2019.

These first-order observations may reflect a multitude of factors, including the composition of researchers in each field, the probability of working on COVID-19, etc. To address this issue, we also employ a Lasso regression approach to select features that are most predictive of the changes in new projects with controlling for other factors. The regression results are reported in Fig. 1ef of the main text and Supplementary Note 7.2.

7.2 Robustness checks using alternative measures

In our main analyses, we use the percentage change in the number of new projects, comparing 2020 with 2019, as the primary measurement of changes in projects. Here we show some robustness checks on the results of group-level and field-level differences by using alternative calculation methods for the changes. First, we calculate the percentage changes in projects using logged values, where $\log(x+1)$ is used to avoid 0 in the raw value x (i.e., the number of new projects). Supplementary Figure 7 shows the average changes and the Lasso regression results based on the percentage changes in logged values. We find that the non-COVID-19 dummy, female, and having young dependents are still features that are most predictive of the declines in new projects (Supplementary Figure 7ab). The changes associated with research fields are relatively small comparing with the sample average, and only the biochemists reported significantly larger declines conditional on other factors (Supplementary Figure 7cd). These observations are highly consistent with the results reported in Fig. 1ef of the main text.

We also repeat our analyses using a measure of absolute changes in raw values from 2019 to 2020 (Supplementary Figure 7e-h). We find that the results remain largely consistent, with only one additional demographic feature selected by the Lasso approach, i.e., having 6-11 years old dependents together with having 0-5 years old dependents (Supplementary Figure 7f). Overall, these results support the robustness of our findings that the effects of losing new projects apply almost universally across fields but split sharply along some demographic dimensions.

7.3 Changes in new collaborators

We analyzed the changes in new collaborators based on the January 2021 survey response (Supplementary Figure 8). We find that scientists reported a large decline in new collaborators in 2020. While only about 15% of scientists reported no new collaborators in 2019, but this fraction becomes more than twice as large at about 35% for 2020 (Supplementary Figure 8a). On average, scientists reported about 3.9 new collaborators in 2019, while this number reduces to about 2.9 in

2020 (Supplementary Figure 8b). The distribution of absolute changes in new collaborators is slightly left-shifted, with a mean and median value being around -1, suggesting a loss of one new collaborator on average in 2020. In a relative term, the decline in new collaborators is more striking, showing a percentage change of -17% on average. These observations are robust when we calculate changes in new collaborators based on logged values.

Moreover, we find that the rate of new collaborators differs massively, however, between COVID scientists (i.e., those who worked on COVID-19-related research) and non-COVID scientists (i.e., those who did not pursue COVID-19-related research) (Supplementary Figure 9). Specifically, COVID scientists reported a similar number of new collaborators in 2019 and 2020, while non-COVID scientists reported a substantial decline that the average number of new collaborators halves from about 4.4 in 2019 to about 2.2 in 2020 (Supplementary Figure 9a). There is a clear left shift in the distribution of absolute changes in new collaborators for non-COVID scientists, with a large negative mean value of -1.5 (Supplementary Figure 9b). By comparison, the distribution for COVID scientists is symmetrical, with a slightly positive mean value. In a relative term, non-COVID scientists reported about 32% reduction in new collaborators in 2020 (Supplementary Figure 9c), while COVID scientists reported a net increase in new collaborators in 2020, with an average change of about 15% above the 2019 level (Supplementary Figure 9d).

7.4 Associations between new projects and new collaborators

We use an OLS regression model to examine the associations between new projects and new collaborators. Supplementary Table 1 summarizes the regression results, where we regress the number of new projects against the number of collaborators with controlling for professional and demographic variables and research fields. We find that the number of new collaborators has a significantly positive effects on the number of new projects even conditional on other important factors. We repeat this regression analysis by using different measures and find the results are robust. Specifically, the absolute changes in new projects are positively associated with the absolute changes in new collaborators, and the percentage changes in new projects are positively associated with the percentage changes in new collaborators (Supplementary Table 2), showing consistent evidence that new projects and new collaborators are significantly and positively associated conditional on other important factors.

Supplementary Note 8. Results based on the Dimensions Publication Data

8.1 Trends of total and average publications per year

We analyze publication trends based on the large-scale publication data. Supplementary Figure 10ab shows the total number of papers published in 2019 and 2020 as tracked by the Dimensions database. We find that the volume of publications increases in 2020 compared with 2019 for both articles (Supplementary Figure 10a) and preprints (Supplementary Figure 10b). This trend remains after excluding COVID-19-related publications in 2020, suggesting that scientists as a whole still published more non-COVID-19 articles and preprints in 2020 than in 2019.

When considering the change in research output at the individual level, however, the data shows a consistent trend with our survey results. Supplementary Figure 10c shows the average number of articles in 2019 and 2020 separated by COVID authors (i.e., those who published COVID-19-related articles in 2020) and non-COVID authors (i.e., those who didn't publish COVID-19-related articles in 2020). We find that COVID authors on average published more articles in 2020 than in 2019, while for non-COVID authors the average number of articles in 2020 is very close to that in

2019. To align with survey respondents who are faculty or principal investigators, we further restrict the analyses for more active and senior authors who published at least one paper per year during the past five years (2015-2019). We find that COVID authors still published more articles in 2020, but non-COVID authors published slightly less articles in 2020 on average compared with 2019 (Supplementary Figure 10d), which is consistent with our observations from the January 2021 survey that non-COVID scientists (i.e., those who worked on COVID-19 research in 2020) reported modest declines in the number of new submissions and publications in 2020 compared with 2019. Further, we vary the time window from five years to one year (Supplementary Figure 10e) and ten years (Supplementary Figure 10f), finding that the results are robust.

8.2 Changes in new co-authorships measured by publication records

Using the Dimensions publication data, we further calculate the fraction of new co-authorships for peer-reviewed articles and preprints published in the past decades (2010-2020). For the year 2020, we further separate the calculation by COVID-19-related papers and non-COVID-19-related papers (Supplementary Figure 11a). We find that the curves are relatively flat from 2010 to 2019 for both articles and preprints. At the same time, there is a notably decrease from 2019 to 2020 for non-COVID-19-related papers and a substantial increase for COVID-19-related papers, showing a distinguish pattern in 2020.

We further calculate the relative ratio of the fractions of new co-authorships for non-COVID-19 preprints comparing the later year with the former year by month for two successive years (Supplementary Figure 11b), e.g., dividing the fraction of 2020 by that of 2019. We find that the ratios for 2019/2018 and 2018/2017 remain largely stable across the year with a value being slightly larger than 1, indicating a minor yet stable increase in new co-authorships over time. Notably, only the 2020/2019 ratio shows a clear decreasing trend during the second half of the year, suggesting a substantial decline in new co-authorships on non-COVID-19 preprints. Furthermore, considering the fraction of new co-authorships can be affected by team size, we separate the analyses by the number of authors on a paper (Supplementary Figure 12). We find that our observations are largely robust across different group sizes, and the effects tend to be more pronounced for small groups compared with large groups.

The Dimensions data also allows us to do cross-country comparisons. According to the information obtained from the World Bank website[19], we identify two groups of countries by their level of income and development using two methods: (1) high income versus low-income countries, and (2) OECD versus least developed countries. We then identify the countries of a paper by the Global Research Identifier Database (GRID) of author’s institutions and calculate the changes in new co-authorships using papers published by these two groups of countries. We find that the effect of decreased new co-authorships on non-COVID-19 publications appears much more pronounced in low-income (least developed) countries than in high-income (OECD) countries (Supplementary Figure 13). Also, the increase of new co-authorships on COVID-19 publications is much smaller in low-income (least developed) countries than in high-income (OECD) countries (Supplementary Figure 13). We find that these results remain robust whether using papers published by a single country or using papers published by one or more countries. Taken together, these results offer preliminary evidence for country-level heterogeneity, where low-income or least developed countries may have been more negatively affected by the pandemic in terms of the rates of new co-authorships, further suggesting the importance of extending our survey framework to more regions of the world.

Supplementary Note 9. Survey Data on COVID-19 Research

9.1 COVID-19-related research by survey data

A growing number of COVID-19-related publications indicate the strong response across the scientific community to the pandemic[20,21]. According to the January 2021 survey, about 34% of survey respondents reported working on COVID-19-related research topics in 2020. We further explore the differences in worked on COVID-19-related research across professional and demographic dimensions and research fields. Supplementary Figure 14 shows the fraction of respondents that worked on COVID-19-related research in 2020. We find that there are relatively small variations across professional and demographic dimensions compared with the sample average (Supplementary Figure 14a). By comparison, there are notable differences across research fields, where a larger fraction of both social and clinical scientists but a small fraction of physicists worked on COVID-19-related research (Supplementary Figure 14b).

We further use a probit regression model, which regress worked on COVID-19-related research in 2020 against professional and demographic variables and the research field dummy (Supplementary Figure 15), finding our conclusions remain robust. Together, these results indicate that whether worked on COVID-19-related research is largely associated with fields rather than with professional and demographic factors such as gender, parenthood, and tenure status.

Supplementary Figures

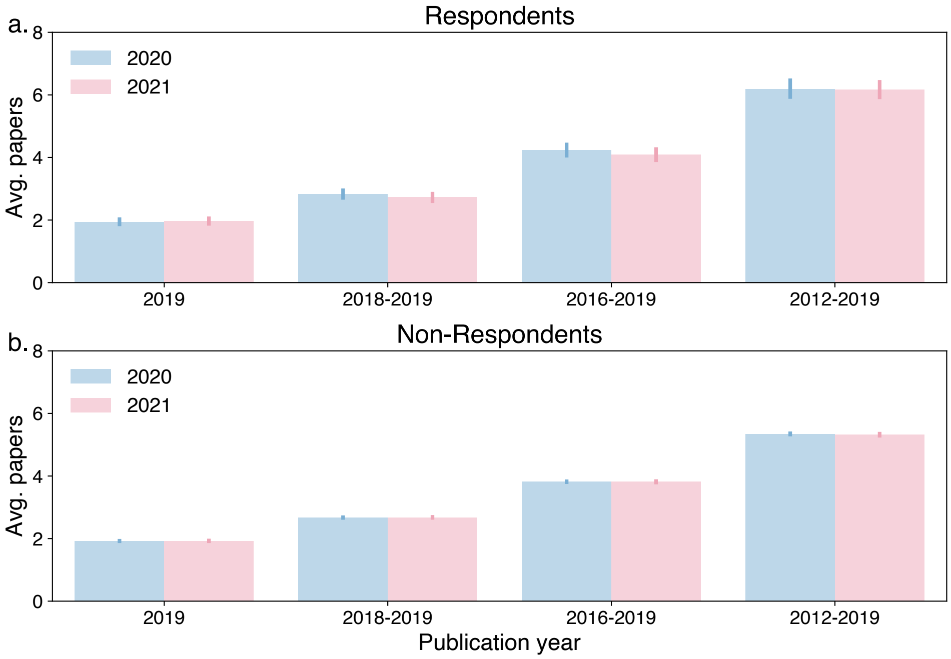

Supplementary Figure 1. Comparing the two survey samples according to publication rates. (a) The average number of papers in the periods of 2019, 2018-2019, 2016-2019, and 2012-2019 for respondents in the April 2020 survey and the January 2021 survey. (b) The average number of papers for non-respondents in the two surveys across the same periods as panel (a). Error bars indicate 95% confidence intervals.

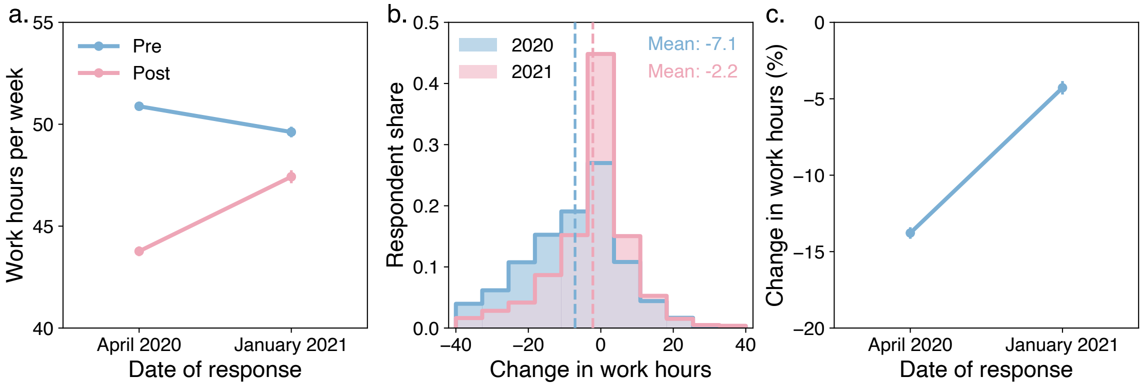

Supplementary Figure 2. Change in total work time. (a) The total works hours per week in pre and post periods across two surveys in April 2020 and January 2021, respectively. (b) The change in total work hours per week comparing post period with pre period in April 2020 and January 2021 surveys. The dashed vertical lines mark the means. (c) The average percentage change in total work hours per week across two surveys. Error bars indicate standard errors.

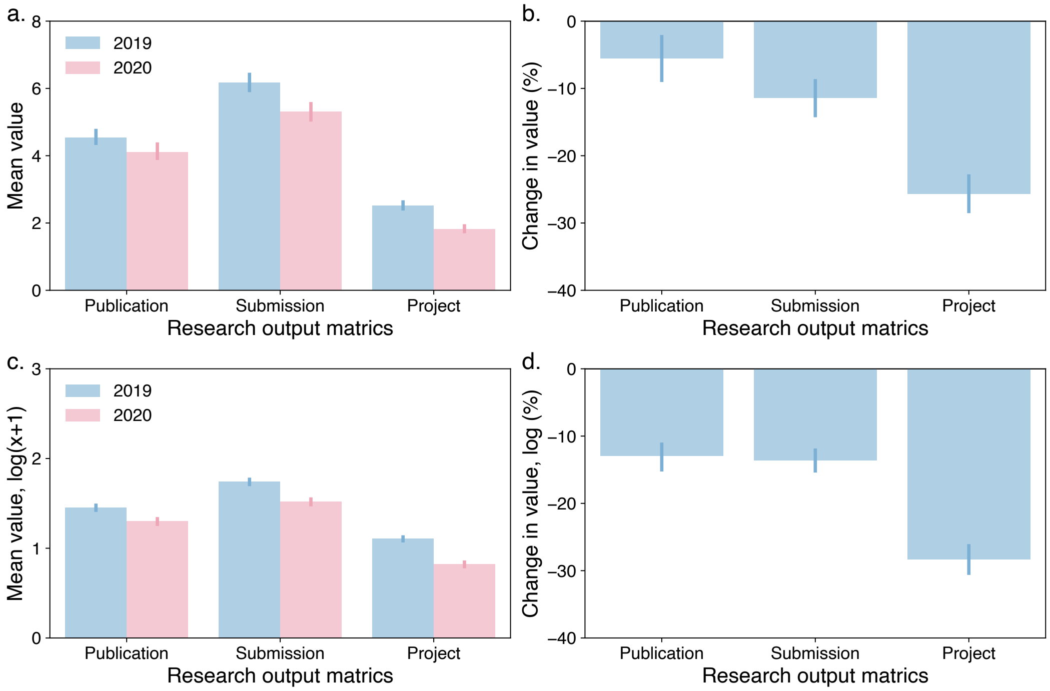

Supplementary Figure 3. Relative changes in research output metrics. (a) The average number of new publications, new submissions, and new projects in 2019 and 2020, respectively. (b) The average percentage change in publications, submissions, and projects, comparing 2020 with 2019 at the individual level. (c-d) The results based on logged values. The raw value x is logged by $\log(x+1)$ to avoid 0. Error bars indicate 95% confidence intervals.

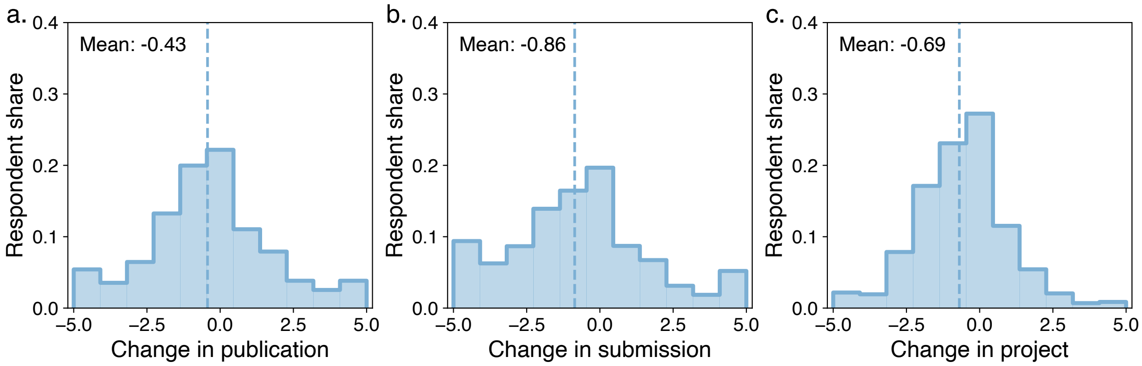

Supplementary Figure 4. Absolute changes in research output metrics. The change in the number of (a) new publications, (b) new submissions, and (c) new projects, comparing 2020 with 2019. Changes that are below -5 or above 5 are set as -5 and 5, respectively. Vertical dashed line indicates the mean change.

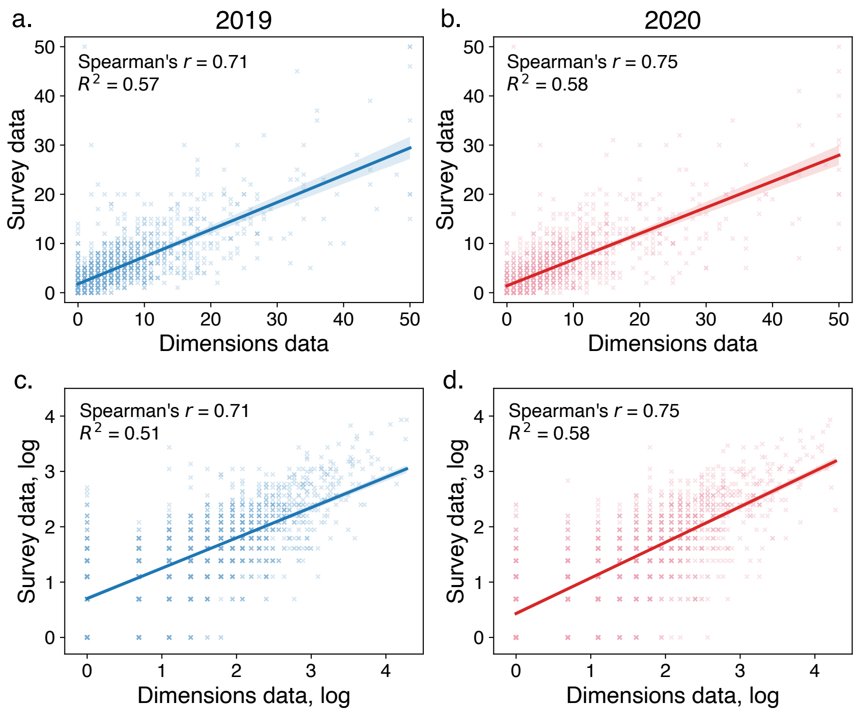

Supplementary Figure 5. Comparison on publications between survey data and Dimensions data for matched scientists. (a) The Spearman's correlation between the numbers of publications for 2019. (b) The Spearman's correlation between the numbers of publications for 2020. (c-d) show results based on logged values. Solid lines indicate linear fits with 95% confidence intervals. The goodness of fit is shown by R2.

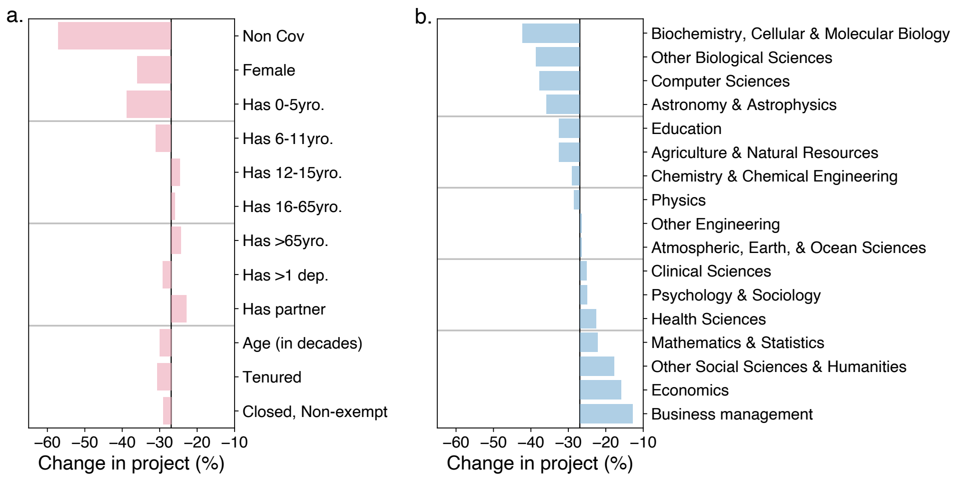

Supplementary Figure 6. Group-level and field-level changes in new projects comparing 2020 with 2019. (a) The percentage changes in new projects, aggerated by professional and demographic dimensions. The non-COVID-19 dummy takes 1 if scientists didn't work on COVID-19-related topics in 2020 and 0 if otherwise. (b) The percentage changes in new projects, aggerated by research fields. The changes are centered by the mean.

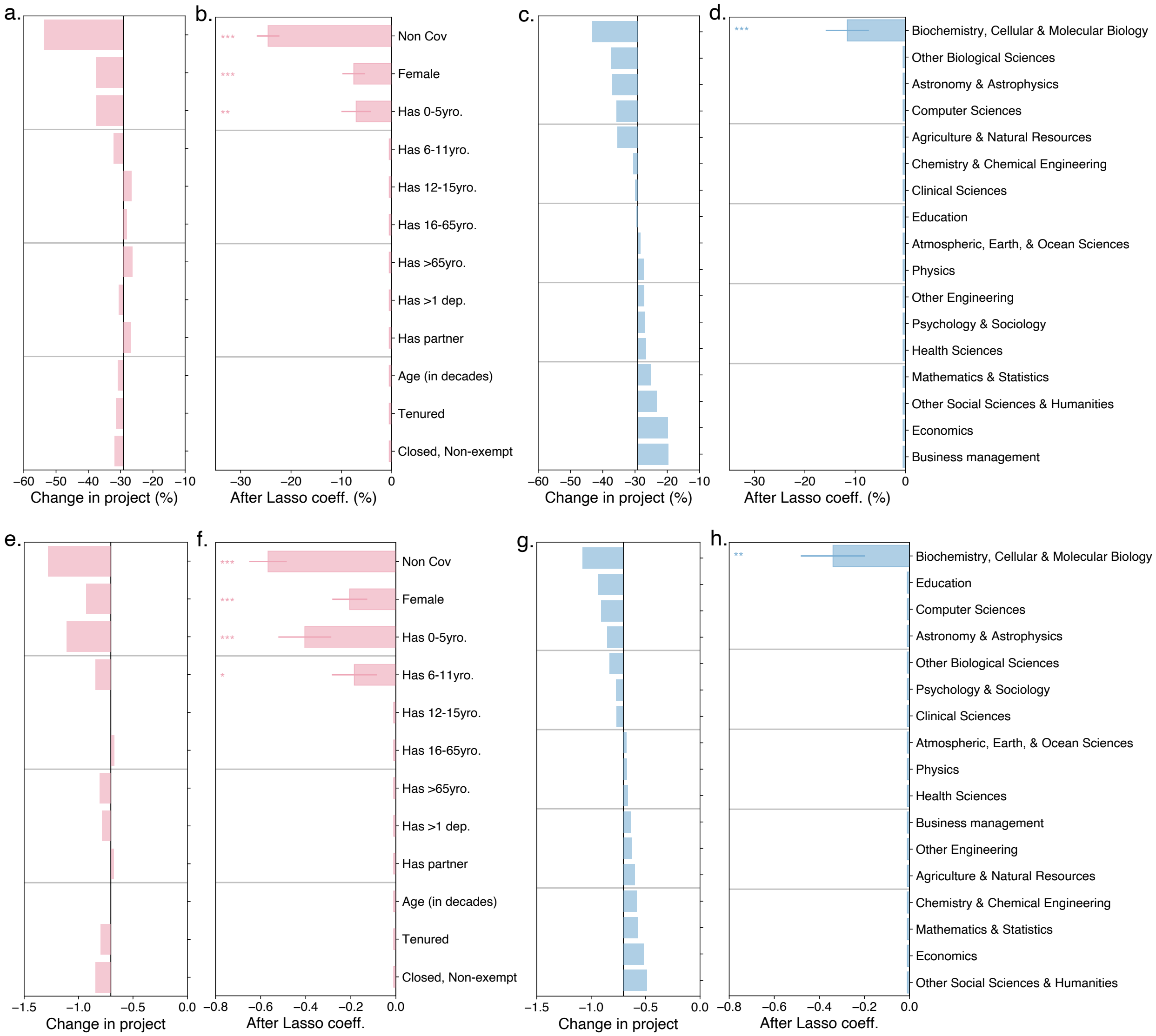

Supplementary Figure 7. Group-level and field-level changes in new projects based on logged and absolute values. (a) The percentage changes in new projects that are aggerated by professional and demographic dimensions. The changes are centered by the mean value. (b) The Lasso regression selects professional and demographic features most predictive of the declines in new projects after controlling for research fields. The regression also includes a non-COVID-19 dummy variable that takes 1 if scientists didn't work on COVID-19-related topics in 2020 and 0 if otherwise. (c) The percentage changes in new projects that are aggerated by research fields. The changes are centered by the mean value. (d) The Lasso regression selects field features most predictive of the declines in new projects after controlling for demographic factors and the non-COVID-19 dummy. (e-h) show the results based on the differences in raw values between 2020 and 2019. Error bars indicate standard errors, and stars indicate significant levels: *p<0.1, **p<0.05, ***p<0.01.

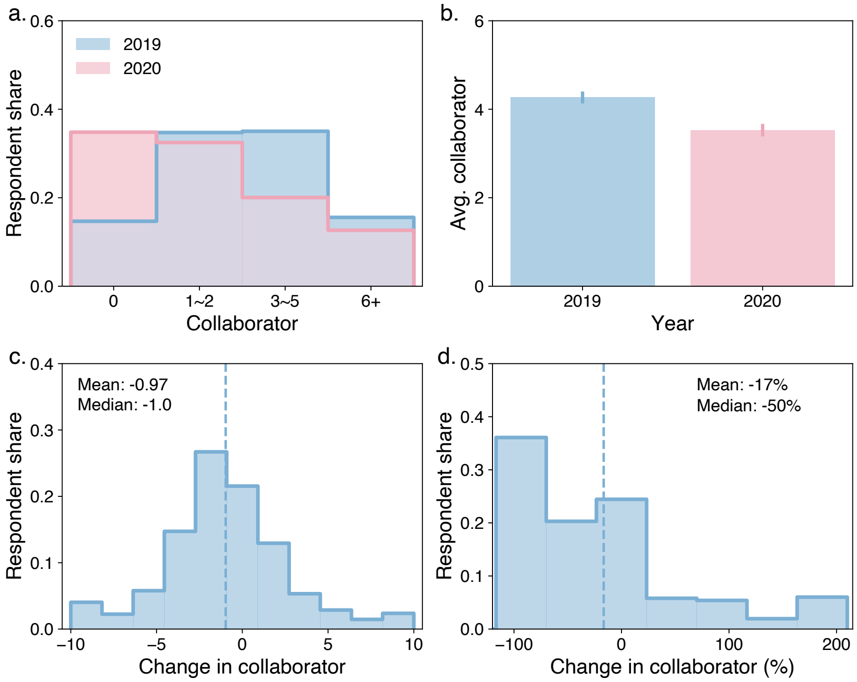

Supplementary Figure 8. Changes in new collaborators measured by survey responses. (a) The distributions of new collaborators in 2019 and 2020 based on the January 2021 survey. (b) The average number of new collaborators in 2019 and 2020. (c) The absolute changes in new collaborators comparing 2020 with 2019. Changes that are below -10 and above 10 are set as -10 and 10, respectively. (d) The distributions of the percentage changes in new collaborators. Changes over 200% are set as 200%.

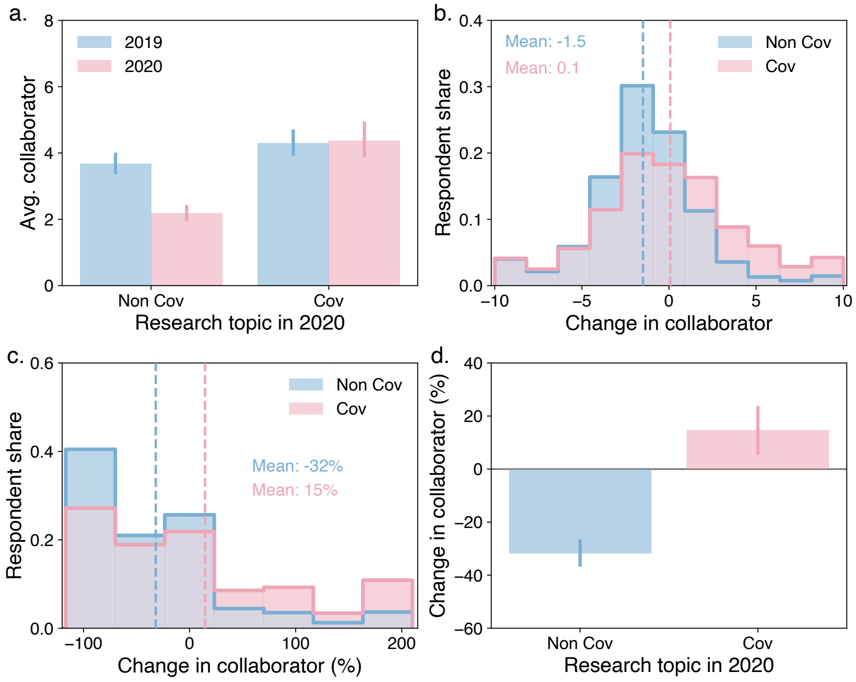

Supplementary Figure 9. Changes in new collaborators for COVID-19 and non-COVID-19 scientists measured by survey responses. (a) The average number of new collaborators in 2019 and 2020. Error bars indicate 95% confidence intervals. (b) The absolute changes in new collaborators comparing 2020 with 2019. Changes that are below -10 and above 10 are set as -10 and 10, respectively. (c) The distributions of the

percentage changes in new collaborators. Changes over 200% are set as 200%. Vertical dashed lines mark the means. (d) The average change in the number of new collaborators in 2020 compared with 2019 for COVID-19 and non-COVID-19 scientists. Throughout the paper, “COVID-19 scientists” indicate those who worked on COVID-19-related research in 2020, and “non-COVID-19 scientists” indicate those who did not pursue COVID-19-related research in 2020. Error bars indicate 95% confidence intervals.

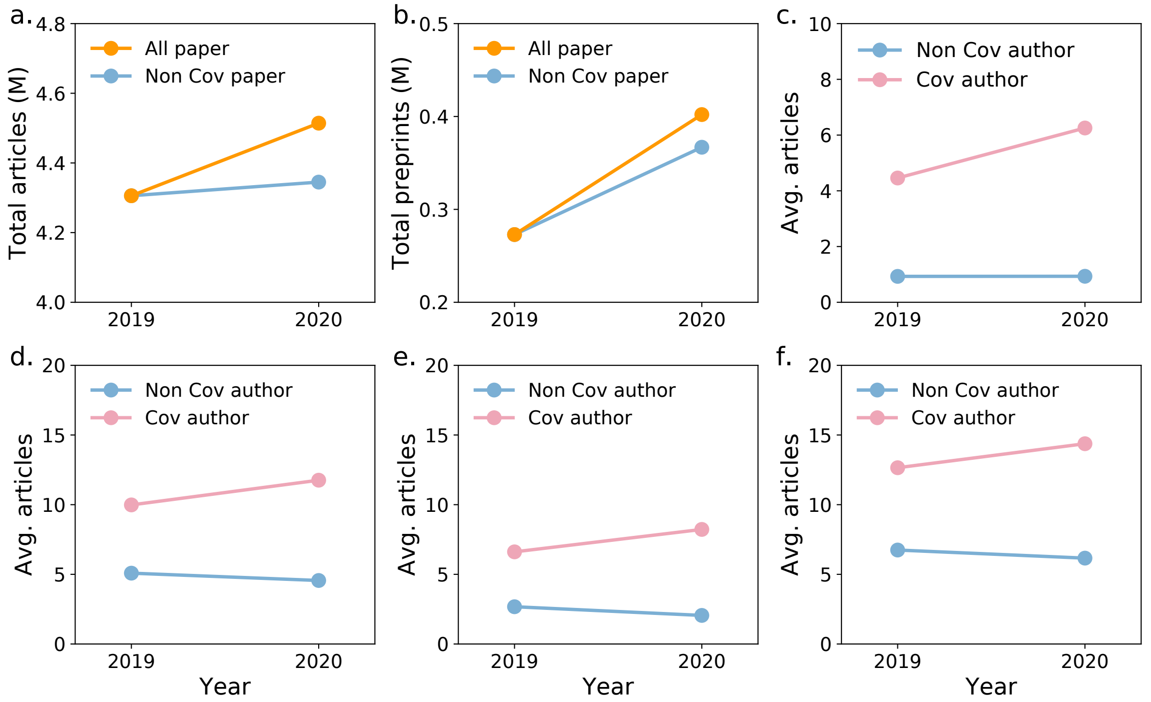

Supplementary Figure 10. The total and average number of publications in 2019 and 2020 according to the Dimensions data. (a) The total number of all articles and non-COVID-19 articles. The gap indicates the number of COVID-19-related articles published in 2020. (b) The total number of all preprints and non-COVID-19 preprints. The gap indicates the number of COVID-19-related preprints published in 2020. (c) The average number of articles in 2019 and 2020 for COVID-19 and non-COVID-19 authors at the individual level for the full sample. (d) Results for authors that published at least one article per year during 2015-2019. (e) Results for authors that published at least one article in 2019. (f) Results for authors that published at least one article per year during 2010-2019. Throughout the paper, “COVID-19 authors” indicate those who published COVID-19-related papers, and “non-COVID-19 authors” indicate those who did not publish COVID-19-related papers.

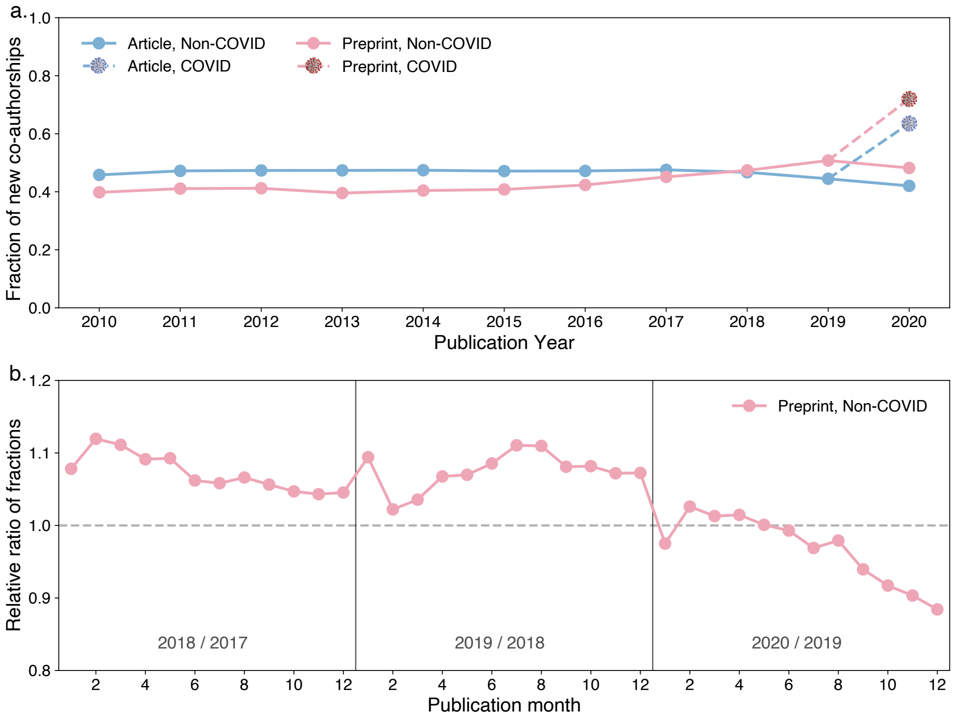

Supplementary Figure 11. The changes in new co-authorships based on publication records. (a) The temporal changes in the fraction of new co-authorships for papers published in a period of 2010-2020. The results are grouped by peer-reviewed articles (in blue) and preprints (in pink). The results are further separated by whether authors published COVID-19-related papers in 2020, namely, non-COVID-19 authors (solid line) and COVID-19 authors (dashed line). (b) The relative ratio of the fractions of new co-authorships in non-COVID-19 preprints published in two successive years. The results show three comparisons in the rate of new co-authorships, namely, 2018 vs 2017, 2019 vs 2018, and 2020 vs 2019.

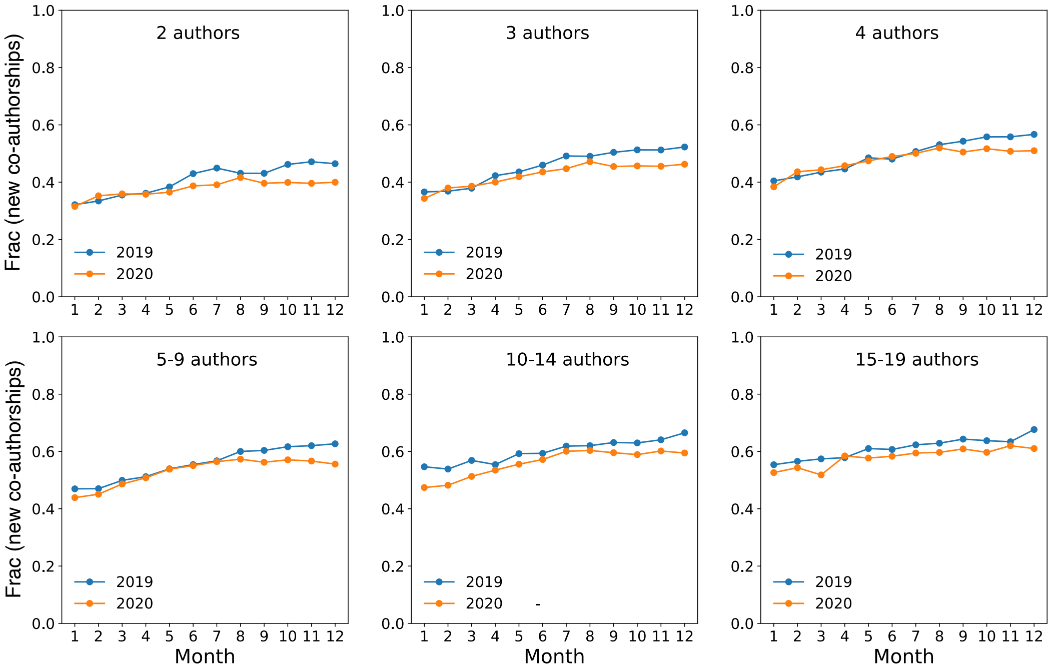

Supplementary Figure 12. The fraction of new co-authorships measured by month for preprints published in 2019 and 2020. The panels show the results for different group sizes, i.e., the number of authors on a preprint.

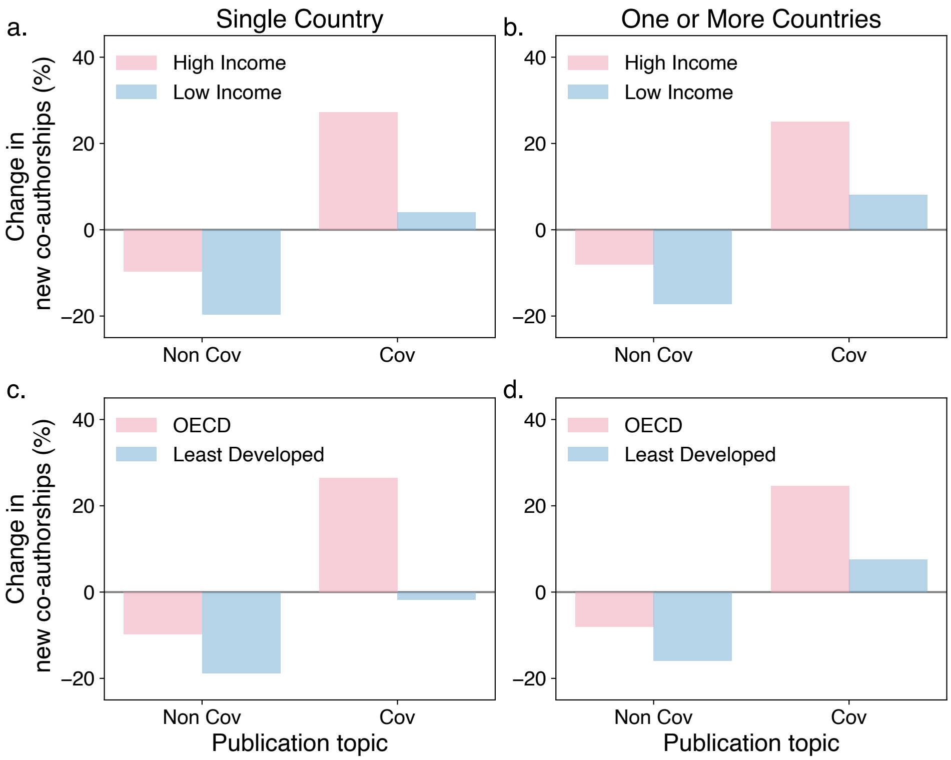

Supplementary Figure 13. Changes in new co-authorships for preprints published in 2019 and 2020 by countries of different income levels. (a) Results based on papers published by a single high-income or low-income country, capturing within-country co-authorships. (b) Results based on papers published by one or more high-income or low-income countries, capturing both within- and cross-country co-authorships. (c) Results based on papers published by an OECD or least developed country. (d) Results based on papers published by one or more OECD or least developed countries.

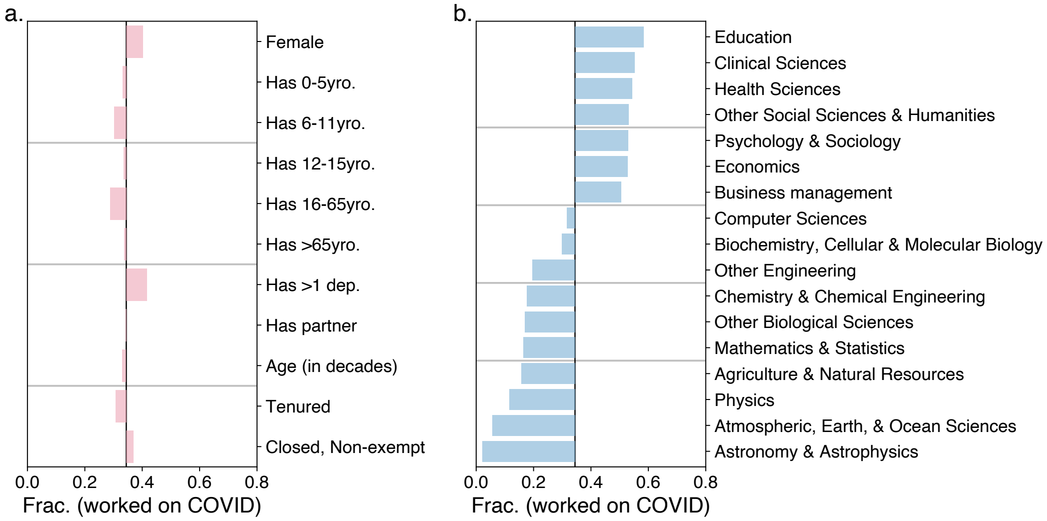

Supplementary Figure 14. The fraction of respondents that worked on COVID-19-related research in 2020. (a) Results aggregated by professional and demographic dimensions. (b) Results aggregated by research fields. The faction is centered by the mean values.

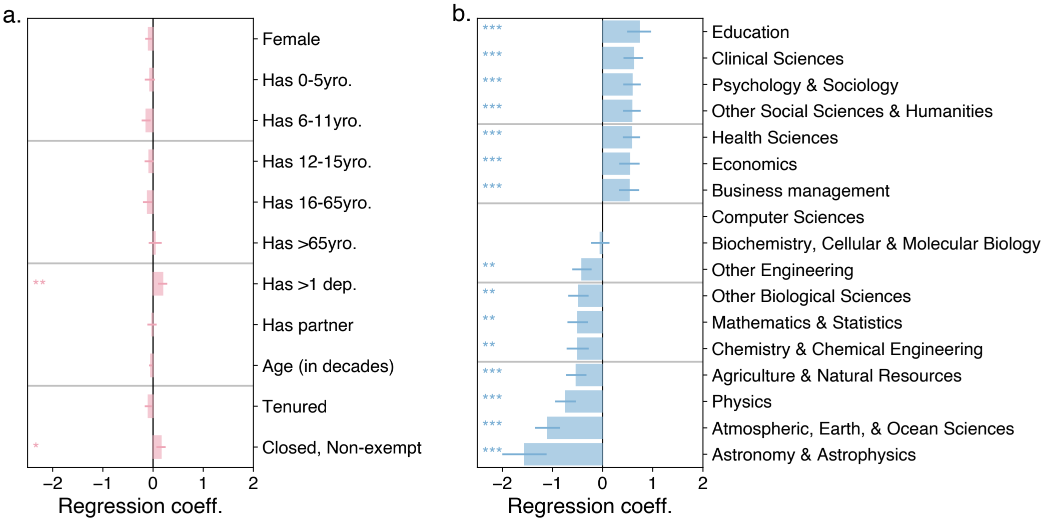

Supplementary Figure 15. Results of a probit regression that predicts whether scientists worked on COVID-19-related research in 2020. (a) Regression coefficients of professional and demographic variables. (b) Regression coefficients of fields. The field "computer sciences" is used as the treatment when including the field dummy in the model. Error bars indicate standard errors, and stars indicate significant levels: $^{*}p<0.1$, $^{**}p<0.05$, $^{***}p<0.01$.

Supplementary Tables

Supplementary Table 1. Results of regressions on the number of new projects against the number of new collaborators with controlling for professional and demographic variables and research fields. The non-COVID-19 dummy takes 1 if scientist haven't worked on COVID-19-related research in 2020 and 0 if otherwise. Robust standard errors in parentheses. Significant levels: *p<0.1, **p<0.05, ***p<0.01.

Variables	Dependent variable: Number of projects									
	2019 raw value		2019 logged value		2020 raw value			2020 logged value		
	(1)	(2)	(3)	(4)	(5)	(6)	(7)	(8)	(9)	(10)
Number of collaborators	0.060***	0.060***	0.226***	0.233***	0.133***	0.118***	0.119***	0.347***	0.312***	0.321***
	(0.016)	(0.016)	(0.017)	(0.018)	(0.021)	(0.021)	(0.021)	(0.016)	(0.016)	(0.016)
Non Cov						-0.878***	-0.850***		-0.254***	-0.238***
						(0.106)	(0.109)		(0.026)	(0.028)
Female		-0.363***		-0.069***			-0.401***			-0.104***
		(0.135)		(0.023)			(0.098)			(0.026)
Has 0-5yro.		0.118		0.075**			-0.019			-0.020
		(0.264)		(0.038)			(0.147)			(0.040)
Has 6-11yro.		0.083		-0.011			-0.063			-0.012
		(0.170)		(0.032)			(0.138)			(0.034)
Has 12-15yro.		-0.053		0.008			0.117			0.033
		(0.219)		(0.034)			(0.139)			(0.035)
Has 16-65yro.		0.073		0.008			0.239*			0.036
		(0.165)		(0.033)			(0.135)			(0.035)
Has >65yro.		0.148		0.000			-0.129			-0.010
		(0.236)		(0.050)			(0.163)			(0.049)
Has >1 dep.		0.128		0.013			-0.128			-0.018
		(0.183)		(0.033)			(0.128)			(0.036)
Has partner		0.064		0.000			0.018			0.018
		(0.149)		(0.033)			(0.147)			(0.036)
Age (in decades)		-0.179***		-0.040***			-0.113**			-0.029**
		(0.057)		(0.012)			(0.054)			(0.013)
Tenured		-0.118		0.001			-0.151			-0.045
		(0.148)		(0.026)			(0.111)			(0.028)
Closed, Non-exempt		0.194		-0.007			-0.060			0.002
		(0.271)		(0.034)			(0.117)			(0.036)
Constant	2.307***	2.813***	0.830***	0.871***	1.417***	2.035***	2.591***	0.500***	0.699***	0.764***
	(0.077)	(0.404)	(0.024)	(0.086)	(0.056)	(0.103)	(0.390)	(0.018)	(0.027)	(0.097)
Dummy (Field)	No	Yes	No	Yes	No	No	Yes	No	No	Yes
Observations	2,074	2,074	2,074	2,074	2,074	2,074	2,074	2,074	2,074	2,074
F	14.76	4.288	169.0	12.24	39.59	62.61	7.754	474.3	313.9	28.54
Adjust R2	0.017	0.034	0.105	0.148	0.100	0.134	0.152	0.223	0.258	0.284
RMSE	2.572	2.549	0.502	0.490	2.109	2.069	2.047	0.554	0.542	0.532

Supplementary Table 2. Results of regressions on the absolute and percentage changes in new projects against the corresponding changes in new collaborators with controlling for professional and demographic variables and research fields. The non-COVID-19 dummy takes 1 if scientist haven't worked on COVID-19-related research in 2020 and 0 if otherwise. Robust standard errors in parentheses. Significant levels: *p<0.1, **p<0.05, ***p<0.01.

Variables	Dependent variable: Change in projects (2020 vs 2019)									
	Absolute change					Percentage change				
	(1)	(2)	(3)	(4)	(5)	(6)	(7)	(8)	(9)	(10)
Change in collaborators	0.107***	0.104***	0.102***	0.101***	0.102***	0.295***	0.279***	0.277***	0.277***	0.278***
	(0.018)	(0.018)	(0.018)	(0.017)	(0.017)	(0.019)	(0.020)	(0.020)	(0.020)	(0.020)
Non Cov		-0.308**	-0.331**	-0.344***	-0.449***		-0.158***	-0.163***	-0.159***	-0.163***
		(0.125)	(0.129)	(0.121)	(0.110)		(0.030)	(0.030)	(0.030)	(0.033)
Female			-0.156	-0.155	-0.057			-0.056**	-0.065**	-0.049
			(0.099)	(0.106)	(0.123)			(0.027)	(0.029)	(0.030)
Has 0-5yro.			-0.376***	-0.193	-0.184			-0.061*	-0.093**	-0.096**
			(0.121)	(0.234)	(0.233)			(0.036)	(0.045)	(0.046)
Has 6-11yro.				-0.177	-0.177				-0.006	-0.007
				(0.157)	(0.157)				(0.039)	(0.039)
Has 12-15yro.				0.161	0.171				0.009	0.005
				(0.182)	(0.185)				(0.039)	(0.039)
Has 16-65yro.				0.181	0.196				0.028	0.030
				(0.142)	(0.140)				(0.040)	(0.040)
Has >65yro.				-0.174	-0.223				0.054	0.049
				(0.199)	(0.206)				(0.055)	(0.056)
Has >1 dep.				-0.199	-0.207				-0.012	-0.013
				(0.166)	(0.160)				(0.039)	(0.040)
Has partner				-0.041	-0.059				0.034	0.035
				(0.140)	(0.139)				(0.037)	(0.037)
Age (in decades)				0.017	0.040				-0.023	-0.021
				(0.046)	(0.048)				(0.015)	(0.015)
Tenured				-0.038	-0.090				-0.053*	-0.065**
				(0.141)	(0.129)				(0.031)	(0.032)
Closed, Non-exempt				-0.264	-0.239				0.021	0.026
				(0.247)	(0.244)				(0.041)	(0.041)
Constant	-0.635***	-0.436***	-0.298**	-0.249	-0.121	-0.240***	-0.138***	-0.102***	0.022	-0.009
	(0.051)	(0.115)	(0.143)	(0.344)	(0.376)	(0.015)	(0.024)	(0.026)	(0.085)	(0.113)
Dummy (Field)	No	No	No	No	Yes	No	No	No	No	Yes
Observations	1,868	1,868	1,868	1,868	1,868	1,868	1,868	1,868	1,868	1,868
F	35.53	22.77	13.94	7.812	4.794	233.0	142.3	75.47	24.82	12.28
Adjust R2	0.0686	0.0720	0.0761	0.0771	0.0788	0.207	0.219	0.221	0.221	0.222
RMSE	2.255	2.251	2.246	2.245	2.242	0.577	0.573	0.572	0.572	0.572

Supplementary References

1 Birkle, C., Pendlebury, D. A., Schnell, J. & Adams, J. Web of Science as a data source for research on scientific and scholarly activity. *Quantitative Science Studies* **1**, 363-376 (2020).
2 Visser, M., van Eck, N. J. & Waltman, L. Large-scale comparison of bibliographic data sources: Scopus, Web of Science, Dimensions, Crossref, and Microsoft Academic. *Quantitative Science Studies* **2**, 20-41 (2021).
3 Wuchty, S., Jones, B. F. & Uzzi, B. The increasing dominance of teams in production of knowledge. *Science* **316**, 1036-1039 (2007).
4 Wang, D. S., Song, C. M. & Barabasi, A. L. Quantifying long-term scientific impact. *Science* **342**, 127-132 (2013).
5 Uzzi, B., Mukherjee, S., Stringer, M. & Jones, B. Atypical combinations and scientific impact. *Science* **342**, 468-472 (2013).
6 Myers, K. R. *et al.* Unequal effects of the COVID-19 pandemic on scientists. *Nature Human Behaviour* **4**, 880-883 (2020).
7 Buuren, S. v. *Flexible imputation of missing data*. Second edition. edn, (CRC Press, Taylor & Francis Group, 2018).
8 Tibshirani, R. Regression shrinkage and selection via the lasso: A retrospective. *Journal of the Royal Statistical Society, Series B (Statistical Methodology)* **73**, 273-282 (2011).
9 Tibshirani, R. Regression shrinkage and selection via the Lasso. *Journal of the Royal Statistical Society: Series B (Methodological)* **58**, 267-288 (1996).
10 Dimensions. <https://www.dimensions.ai> (2021).
11 Yin, Y., Gao, J., Jones, B. F. & Wang, D. Coevolution of policy and science during the pandemic. *Science* **371**, 128-130 (2021).
12 Dimensions Resources. *Dimensions COVID-19 publications, datasets and clinical trials*, <https://dimensions.figshare.com/articles/dataset/Dimensions_COVID-19_publications_datasets_and_clinical_trials/11961063> (2020).
13 Zeng, A., Fan, Y., Di, Z. R., Wang, Y. G. & Havlin, S. Fresh teams are associated with original and multidisciplinary research. *Nature Human Behaviour*, doi:10.1038/s41562-021-01084-x (2021).
14 Guimera, R., Uzzi, B., Spiro, J. & Amaral, L. A. N. Team assembly mechanisms determine collaboration network structure and team performance. *Science* **308**, 697-702 (2005).
15 Milojevic, S. Principles of scientific research team formation and evolution. *Proceedings of the National Academy of Sciences, U.S.A.* **111**, 3984-3989 (2014).
16 Wu, L., Wang, D. & Evans, J. A. Large teams develop and small teams disrupt science and technology. *Nature* **566**, 378-382 (2019).
17 Jones, B. F., Wuchty, S. & Uzzi, B. Multi-university research teams: shifting impact, geography, and stratification in science. *Science* **322**, 1259-1262 (2008).
18 Newman, M. E. J. The structure of scientific collaboration networks. *Proceedings of the National Academy of Sciences, U.S.A.* **98**, 404-409 (2001).
19 The World Bank Group. *World Bank Country and Lending Groups*, <https://datahelpdesk.worldbank.org/knowledgebase/articles/906519-world-bank-country-and-lending-groups> (2021).
20 Van Bavel, J. J. *et al.* Using social and behavioural science to support COVID-19 pandemic response. *Nature Human Behaviour* **4**, 460-471 (2020).
21 Hill, R., Yin, Y., Stein, C., Wang, D. & Jones, B. Adaptability and the pivot penalty in science. SSRN 3886142 (2021).
