# Supplementary material for: Potentially long-lasting effects of the pandemic on scientists
Source: Nat Commun. 2021 Oct 26;12:6188. doi: 10.1038/s41467-021-26428-z (PMC8548590; doi:10.1038/s41467-021-26428-z)
Supplement: Supplementary file 2 — Reporting summary [file 41467_2021_26428_MOESM2_ESM.pdf]

nature research

Corresponding author(s): Dashun Wang

Last updated by author(s): Aug 30, 2021

Reporting Summary

Nature Research wishes to improve the reproducibility of the work that we publish. This form provides structure for consistency and transparency in reporting. For further information on Nature Research policies, see our Editorial Policies and the Editorial Policy Checklist.

Statistics

For all statistical analyses, confirm that the following items are present in the figure legend, table legend, main text, or Methods section.

n/a	Confirmed	
☐	☒	The exact sample size (*n*) for each experimental group/condition, given as a discrete number and unit of measurement
☒	☐	A statement on whether measurements were taken from distinct samples or whether the same sample was measured repeatedly
☒	☐	The statistical test(s) used AND whether they are one- or two-sided<br>*Only common tests should be described solely by name; describe more complex techniques in the Methods section.*
☒	☐	A description of all covariates tested
☒	☐	A description of any assumptions or corrections, such as tests of normality and adjustment for multiple comparisons
☐	☒	A full description of the statistical parameters including central tendency (e.g. means) or other basic estimates (e.g. regression coefficient) AND variation (e.g. standard deviation) or associated estimates of uncertainty (e.g. confidence intervals)
☐	☒	For null hypothesis testing, the test statistic (e.g. *F*, *t*, *r*) with confidence intervals, effect sizes, degrees of freedom and *P* value noted<br>*Give P values as exact values whenever suitable.*
☒	☐	For Bayesian analysis, information on the choice of priors and Markov chain Monte Carlo settings
☒	☐	For hierarchical and complex designs, identification of the appropriate level for tests and full reporting of outcomes
☐	☒	Estimates of effect sizes (e.g. Cohen's *d*, Pearson's *r*), indicating how they were calculated

Our web collection on statistics for biologists contains articles on many of the points above.

Software and code

Policy information about availability of computer code

Data collection	Survey data was collected through Qualtrics, and publication records were collected from the Dimensions database.
Data analysis	Data is analyzed with customized code in Stata 16 and Python 3 using standard software packages within these programs.

For manuscripts utilizing custom algorithms or software that are central to the research but not yet described in published literature, software must be made available to editors and reviewers. We strongly encourage code deposition in a community repository (e.g. GitHub). See the Nature Research guidelines for submitting code & software for further information.

Data

Policy information about availability of data

All manuscripts must include a data availability statement. This statement should provide the following information, where applicable:

- Accession codes, unique identifiers, or web links for publicly available datasets
- A list of figures that have associated raw data
- A description of any restrictions on data availability

Because of the sensitive nature of some of the variables collected through the surveys, the IRB-approved protocol does not permit individual-level data to be made unrestricted and publicly available. Researchers interested in obtaining restricted, anonymized versions of this individual-level data should contact the authors to inquire about obtaining an IRB-approved institutional data sharing agreement. This work also uses data sourced from Web of Science and Dimensions.ai. Researchers who wish to access raw data should contact the data sources directly.

Field-specific reporting

Please select the one below that is the best fit for your research. If you are not sure, read the appropriate sections before making your selection.

- [ ] Life sciences
- [x] Behavioural & social sciences
- [ ] Ecological, evolutionary & environmental sciences

For a reference copy of the document with all sections, see nature.com/documents/nr-reporting-summary-flat.pdf

Behavioural & social sciences study design

All studies must disclose on these points even when the disclosure is negative.

Study description	A study to quantify the impact of COVID-19 pandemics on scientists.
Research sample	We identified scientists in US and Europe with at least two scientific papers during the past decade. Further details are available in Supplementary Note 1.
Sampling strategy	We collected a list of author email addresses from Web of Science. We then randomly shuffled and sampled roughly 280,000 email addresses from U.S.-based authors and 200,000 from Europe-based authors for the April 2020 survey. From remaining email addresses, we randomly sampled 140,000 from the U.S. and 100,000 from Europe for the January 2021 survey. Further details are available in Supplementary Note 1.
Data collection	We sent out email invitations with a link to an online survey form. The survey is hosted and collected through the Qualtrics platform.
Timing	The two surveys were performed in April 2020 and January 2021, respectively.
Data exclusions	For our analyses, we focus entirely on responses from the sample of faculty/Principal Investigators, excluding responses from individuals who report to work for a “For-profit firm”. We restrict the sample to respondents whose IP address originated from the United States or Europe and drop observations that have missing data for the variables used in our analyses. Further details are available in Supplementary Note 2.
Non-participation	We estimate a response rate of approximately 1.6% for the April 2020 survey and 1.9% for the January 2021 survey. Further details are available in Supplementary Note 2.
Randomization	There is no randomization related to used survey questions across the two surveys.

Reporting for specific materials, systems and methods

We require information from authors about some types of materials, experimental systems and methods used in many studies. Here, indicate whether each material, system or method listed is relevant to your study. If you are not sure if a list item applies to your research, read the appropriate section before selecting a response.

Materials & experimental systems

n/a	Involved in the study	
x		Antibodies
x		Eukaryotic cell lines
x		Palaeontology and archaeology
x		Animals and other organisms
	x	Human research participants
x		Clinical data
x		Dual use research of concern

Methods

n/a	Involved in the study	
x		ChIP-seq
x		Flow cytometry
x		MRI-based neuroimaging

Human research participants

Policy information about studies involving human research participants

Population characteristics	See above.
Recruitment	We recruit individuals online through sending invitation emails. Further details on representativeness of our sample are available in Supplementary Notes 1-2.
Ethics oversight	The study protocol was approved by the IRB from Northwestern University and Harvard University.

Note that full information on the approval of the study protocol must also be provided in the manuscript.
